# Neurovirulence of H5N1 Infection in Ferrets Is Mediated by Multifocal Replication in Distinct Permissive Neuronal Cell Regions

**DOI:** 10.1371/journal.pone.0046605

**Published:** 2012-10-08

**Authors:** Jennifer R. Plourde, John A. Pyles, R. Colby Layton, Sarah E. Vaughan, Jennifer L. Tipper, Kevin S. Harrod

**Affiliations:** 1 Infectious Diseases Program, Lovelace Respiratory Research Institute, Albuquerque, New Mexico, United States of America; 2 Department of Pathology, University of New Mexico School of Medicine, Albuquerque, New Mexico, United States of America; University of Georgia, United States of America

## Abstract

Highly pathogenic avian influenza A (HPAI), subtype H5N1, remains an emergent threat to the human population. While respiratory disease is a hallmark of influenza infection, H5N1 has a high incidence of neurological sequelae in many animal species and sporadically in humans. We elucidate the temporal/spatial infection of H5N1 in the brain of ferrets following a low dose, intranasal infection of two HPAI strains of varying neurovirulence and lethality. A/Vietnam/1203/2004 (VN1203) induced mortality in 100% of infected ferrets while A/Hong Kong/483/1997 (HK483) induced lethality in only 20% of ferrets, with death occurring significantly later following infection. Neurological signs were prominent in VN1203 infection, but not HK483, with seizures observed three days post challenge and torticollis or paresis at later time points. VN1203 and HK483 replication kinetics were similar in primary differentiated ferret nasal turbinate cells, and similar viral titers were measured in the nasal turbinates of infected ferrets. Pulmonary viral titers were not different between strains and pathological findings in the lungs were similar in severity. VN1203 replicated to high titers in the olfactory bulb, cerebral cortex, and brain stem; whereas HK483 was not recovered in these tissues. VN1203 was identified adjacent to and within the olfactory nerve tract, and multifocal infection was observed throughout the frontal cortex and cerebrum. VN1203 was also detected throughout the cerebellum, specifically in Purkinje cells and regions that coordinate voluntary movements. These findings suggest the increased lethality of VN1203 in ferrets is due to increased replication in brain regions important in higher order function and explains the neurological signs observed during H5N1 neurovirulence.

## Introduction

Highly pathogenic avian influenza A (HPAI), subtype H5N1, has infected humans in 12 countries and has been associated with approximately a 60% mortality rate since 1997 (http://www.who.int/influenza/human_animal_interface/EN_GIP_20111010CumulativeNumberH5N1cases.pdf). Severe disease of H5N1 includes fast-progressing pneumonia, acute respiratory distress syndrome (ARDS), diarrhea, central nervous system (CNS) clinical signs, and multi-organ failure. Death often occurs within ten days of symptom onset [1]–[3]. Studies to identify virulence factors contributing to these phenotypes have been the focus of many recent investigations [4]–[8]. However the mechanisms leading to increased pathogenesis by H5N1, particularly non-pulmonary events, remain to be elucidated.

ARDS is a common manifestation of pulmonary influenza infection; however H5N1 has been atypically shown to also infect and damage the CNS. De Jong and colleagues reported acute encephalitis in brains of humans infected with H5N1. These patients did not present with respiratory illness but had severe diarrhea, with early onset of seizures and coma, and death occurring within one to five days post hospital admittance [9]. Murine infection models have illustrated that neurotropic H5N1 strains exhibit higher lethality than those that do not replicate efficiently in the brain [10]. Several groups have investigated possible routes of viral entry into the brain, including the olfactory system as a major route into the brain of experimentally infected ferrets [10]–[12]. Studies by Park *et al.* suggested that, in addition to the olfactory nerves, HPAI enters the CNS through the vagal, trigeminal, and sympathetic nerves [12]. Furthermore, the dissemination of H5N1 through the bloodstream is plausible due to the presence of virus in organs such as the spleen apart from the site of initial infection.

While these possible routes of infection in the brain have been identified, little is known regarding HPAI dissemination within the CNS and its contribution to clinical signs and lethality. Therefore, delineating neurotropic features of H5N1 infection in the ferret model could lead to a better understanding of mechanisms responsible for widespread infection throughout the central nervous system.

Herein, we compare two strains of H5N1 with distinct neurotropism and lethality in ferrets to elucidate the temporal-spatial neuroinvasion leading to death. We show that VN1203 resulted in wider dissemination in the brain and associated with higher morbidity and clinical signs of neurological involvement. By comparison, HK483 infection resulted in low mortality, no viable virus recovered from the brain, and a low incidence of brain lesions limited solely to the olfactory system. Furthermore, we identify brain regions and cell types susceptible to VN1203 that explain the myriad of neurological signs during lethal infection. These findings broaden our understanding of the neurovirulence of H5N1 viruses and support further investigation into therapies leading to CNS protection.

## Materials and Methods

### Ethics statement

The protocol (FY10-098) for all animal procedures was approved by the Institutional Animal Care and Use Committee (IACUC) and Institutional Biosafety Committee of the Lovelace Respiratory Research Institute, Albuquerque, NM. All facilities were accredited by the Association for Assessment and Accreditation of Laboratory Animal Care International (AAALAC). Ferret experiments were conducted in the Animal Biosafety Level 3 enhanced (ABSL-3+) facility and guidelines for ferret housing, environment, and comfort described in the Guide For The Care and Use of Laboratory Animals, Seventh Edition, National Research Council, were strictly followed. Euthanasia was performed under the guidance of the American Veterinary Medical Associations (AVMA) Guidelines on Euthanasia.

### Virus preparation

Highly pathogenic avian influenza A (HPAI) H5N1 viruses were obtained from the Centers for Disease Control and Prevention (CDC) (Atlanta, GA). A/Vietnam/1203/2004 (VN1203) was isolated from a pharyngeal swab from a ten year old male patient that died of the disease in Vietnam in 2004 [7] and A/Hong Kong/483/1997 (HK483) was isolated from a 13 year old male patient that died of the disease in Hong Kong in 1997 [7]. These viruses were propagated from the CDC stock in eggs twice to produce working stocks, aliquoted, titrated by plaque assay on Madin-Darby Canine Kidney (MDCK) cells, and stored at −80°C. All manipulations with these viruses were conducted under Biosafety Level 3 (BSL-3) conditions in the BSL-3 or animal BSL-3 enhanced (ABSL-3+) at the Lovelace Respiratory Research Institute in Albuquerque, New Mexico.

### Ferret handling/care and infection

Castrated male ferrets (*Mustela putorius furo*), 11–14 weeks of age at the day of infection, weighing 0.7 to 1.0 kg, (supplied by Triple F Farms, Sayre, PA) were used for the studies. They were pre-screened by the supplier for H1 and H3 influenza A and influenza B seroconversion prior to shipment and similarly screened at LRRI within a week prior to infection. Ferrets were triple-housed throughout (including 14 days of quarantine and after viral challenge) in stainless steel cages and in separate BioBubbles® depending on H5N1 strain. Ferrets had access to food and water *ad libitum*. Temperature and humidity ranged 16 to 22°C and 30 to 65% respectively, and the light cycle was 12 hr on and 12 hr off. Ventilation in the study room was >15 air exchanges per hour. All ferret handlers were vaccinated for circulating seasonal influenza strains and were not permitted to enter ferret quarters if they exhibited any symptoms of upper or lower respiratory infection.

### Viral infection of ferrets

Ferrets were identified by an IPTT-300 Implantable Programmable Temperature and Identification Transponder; Bio Medic Data Systems, Inc, (BMDS) (Seaford, Delaware). These chips also provided subcutaneous body temperature data using a BMDS electronic proximity reader wand (WRS-6007; BMDS). Ferrets were anesthetized with an intramuscular injection of a ketamine/xylazine cocktail (20 mg/kg of ketamine and 2 mg/kg of xylazine). Ferrets received 0.500 mL of pathogen or allantoic fluid per naris with a total of 1.0 mL delivered. Each subject was held upright for about one minute to ensure retention of the inoculum. Groups of ten ferrets were instilled with 10 PFU of VN1203 or HK483. An additional group of 18 ferrets was also infected with 10 PFU of HK483 and groups of six were sacrificed 4, 6, and 8 days post-infection for examination by virology and immunohistochemical assays.

Clinical observations were conducted twice daily and included temperature readings from the BMDS microchip and recording of clinical signs of disease. Study personnel were trained specifically in ferret handling procedures and the observation and recording of adverse signs and clinical symptoms in ferrets. Observations were recorded using an electronic data recording system. The onset, nature, severity, and duration of all visible changes such as abnormal respiration, excretions, behavioral, and neurological signs (*i.e.*, paresis, torticollis, seizures, and paralysis) were recorded as well as any observed coughing, sneezing, and nasal or ocular discharge. Additionally, an activity score was assigned at each observation as follows: (0 = alert and playful, 1 = alert but playful only when stimulated, 2 = alert but not playful when stimulated, and 3 = neither alert nor playful when stimulated).

### Cell culture and infection

Primary differentiated cells were isolated from the nasal turbinates of naïve ferrets and the protocol was adapted with minor modifications as described previously [13]. Cells were rinsed from the tissue and plated directly onto collagen-coated 100 mm tissue culture dishes. Cells were plated directly onto collagen coated transwell inserts (Corning Inc., Lowell, MA) with BEGM plus DMEM (Sigma-Aldrich Co. LLC., St. Louis, MO) media in the basolateral and apical chambers. Approximately seven days later, the media in the apical chamber was removed and the cells were maintained under air-liquid interface conditions. VN1203 and HK483 stocks were diluted in sterile 1× PBS to a multiplicity of infection of 0.001 in 100 µL. A volume of 100 µl of diluted virus or 1× PBS for control was applied to the apical surface of the cells and virus was allowed to penetrate the cells for one hour at 37°C and 5% CO_2_. Following one hour of incubation, the inoculum was removed and cells were returned to the incubator until the next collection. The inoculum was titered to determine if there was a difference in adsorption between the two strains. Approximately 500 FFU/mL was delivered and 0 FFU/mL was recovered from the HK483 infected wells and an average of 5 FFU/mL was recovered from the VN1203 infected wells. Apical washes were collected by adding 200 µL of 1× PBS onto the surface and pipetting five times. This was repeated for a total collection of 400 µL. Apical washes were stored at −80C until viral titers were determined by an immunostaining foci-forming assay.

### Real-time quantitative PCR analysis

Approximately 250 mg of ferret tissue was homogenized in 1 mL of sterile 1× PBS at the time of collection and a 0.200 mL aliquot was added to Trizol® for a final volume of 1 mL. Viral RNA was extracted using the Qiagen RNeasy kit and the Qiagen Universal Robot (Qiagen Inc., Valencia, CA). Amplification of the full viral coding sequence was done as previously described in [14]. All assays were repeated in triplicate on the ABI 7300 real-time PCR system (Applied Biosystems, LifeTechnologies Corp., Carlsbad, CA). The quantitative PCR results were analyzed with the software provided.

### Assay for viral titers

Viral titers from the apical wash of infected primary differentiated nasal turbinate cells and from ferret tissue homogenates were determined using the immunostaining foci-forming assay. Approximately 250 mg tissue sections were removed from the right caudal hilar, right caudal peripheral, left cranial hilar, and left cranial peripheral lung lobes, right nasal turbinate, olfactory bulb, cerebral cortex, and brain stem. These tissues were placed in 1 mL of sterile 1× PBS with one 5 mm stainless steel bead and homogenized with a Qiagen tissuelyser (Qiagen Inc., Valencia, CA) for 2 min at 30 Hz/sec. Viral titers were determined using the 96 well format previously described in [15]. Virus present in the cells was stained with anti-influenza A antibody, MAB-8251, (Millipore, Billerica, MA), an anti-goat secondary antibody (Vector Laboratories Inc., Burlingame, CA), and detected with 3-Amino-9-ethylcarbazole (AEC) (Sigma-Aldrich Co. LLC., St. Louis, MO). Stained foci were counted and the titer was reported as foci-forming units per mL (FFU/mL).

### Hematoxylin and eosin (H&E) stain and immunohistochemistry of ferret tissue sections

Lungs were inflation fixed at 15 cm of pressure with 10% neutral buffered formalin. The skull, lung, and brain were fixed in formalin for seven days then changed to 70% ethanol. Fixed tissues were embedded in paraffin and tissue sections (4–6 µm) were stained by H&E for histopathological examination under light microscopy. Immunohistochemical staining was conducted using standard protocols. Briefly, endogenous peroxidases were blocked and antigen retrieval was performed using a citrate buffer. Avian influenza nucleoprotein was detected using IMG-5187A, (IMGENEX, San Diego, CA) [16], a biotinylated secondary antibody, and the ABC standard and DAB kits for detection (Vector Laboratories Inc., Burlingame, CA). Images were acquired using the Hamamatsu 2.0 NanoZoomer software (Hamamatsu Corp., Bridgewater, NJ). Histological determinations were made by a board-certified veterinary pathologist unfamiliar with the study design.

### Statistical Analysis

GraphPad Prism 5, software version 5.04 (La Jolla, CA) was used to graph data and perform statistical analysis. The Kaplan Meier survival curve was used for the survival analysis, a Student's t-test was used for comparing two means, and an analysis of variance (ANOVA) was used for comparing multiple means. Mean ± SEM is graphed and p≤.05 was considered statistically significant.

## Results

### HPAI lethality and clinical signs

**Figure 1:**
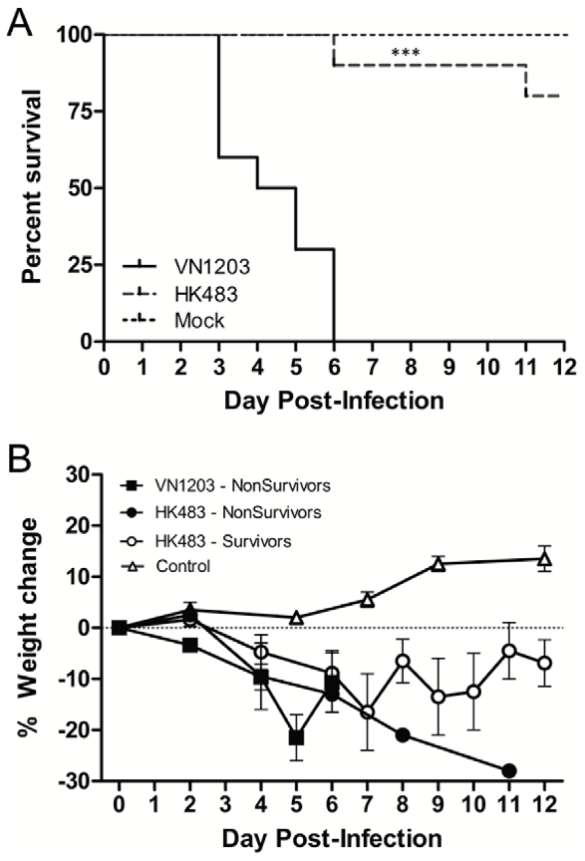
VN1203 was more lethal than HK483 in ferrets despite similar weight loss in non-survivors. **A.** Survival of ferrets intranasally challenged with allantoic fluid (N = 2), A/Vietnam/1203/2004 (VN1203) (N = 10) or A/Hong Kong/483/1997 (HK483) N = 10, ***, p<.0001. **B.** Mean ± SEM of bodyweight of ferrets instilled with VN1203 or HK483. N = 8 HK483 survivors, N = 2 HK483 non-survivors, N = 10 VN1203 non-survivors, N = 2 control.

The neurotropism of HPAI viruses has been reported in human cases as well as in experimental animal models [4], [9], [10], [12], [17]–[21]. Despite these reports, the neuropathogenesis of HPAI is poorly understood. To test the contribution of neurotropism to the lethality of two HPAI strains, low inoculating titers of A/Hong Kong/483/1997 (HK483) and A/Vietnam/1203/2004 (VN1203) were delivered by intranasal instillation to naïve ferrets using a dose of approximately 10 PFU to simulate the low amount of virus likely to be received in a natural infection. As reported previously, VN1203 is known to cause death at lower inoculating titers than HK483 [7] and in this study VN1203 infection resulted in a significantly earlier median time to death (MTTD = 4 dpi) and 100% mortality (*n* = 10) within 6 days post-infection (dpi) whereas infection with 10 PFU of HK483 resulted in only 20% lethality (*n* = 10) (**Figure 1A**; Mantel Cox test, p<.0001, MTTD unable to be determined). Body weight was measured daily in all animals prior to and following infection (**Figure 1B**). Mock infected ferrets maintained body weight or increased over the twelve day study period, while all animals that received either VN1203 or HK483 declined in body weight. Of animals receiving HK483, survivors and non-survivors exhibited similar declines in body weight both in timing and magnitude, indicating progressing disease. Importantly, many ferrets receiving VN1203 succumbed prior to appreciable losses in body weight. There was no significant difference in weight loss post-infection with either VN1203 or HK483 at inoculating titers of 10 PFU and the eight surviving HK483 challenged ferrets began to gain weight by 12 dpi.

To assess clinical signs during infection with the two different strains, cage side observations were performed twice daily on ferrets following 10 PFU of VN1203 or HK483 infection (*n* = 10 per group). Seven ferrets instilled with VN1203 developed nasal discharge 3 dpi with 100% presenting nasal discharge by 4 dpi. Similarly, 100% of HK483 infected ferrets exhibited nasal discharge by 12 dpi with two subjects presenting symptoms as early as 3 dpi. Ocular discharge was noted between 3 and 6 dpi in two ferrets challenged with VN1203 and four ferrets challenged with HK483. These clinical signs indicated upper respiratory tract infection in the nose and conjunctiva. Eight of ten ferrets challenged with VN1203 developed neurological signs with four ferrets observed to have convulsions in the absence of fever 3 dpi, torticollis observed 5 dpi in one ferret, and paresis of musculoskeletal dysfunction noted in three ferrets 6 dpi (**Table 1**). Of the two HK483 challenged ferrets that did not survive the study period, one died 6 dpi due to severe influenza-associated pneumonia and secondary bacterial lung infection as determined by histological examination and the second was humanely euthanized after losing more than 25% of its initial bodyweight by 11 dpi. Collectively, these findings indicated that a low dose of VN1203 produced more neurological signs, earlier time to death, and greater lethality compared to ferrets infected with HK483 while weight loss and symptoms in the respiratory tract were not key factors in lethality caused by VN1203.

**Table 1:** Clinical observations in ferrets post-challenge with VN1203 or HK483.

H5N1 strain	Nasal Discharge	Ocular Discharge	Neurological Symptoms
**VN1203**	10/10	2/10	8/10
**HK483**	10/10	4/10	0/10

Number of ferrets with clinical symptoms (# with symptoms/total # in group).

### HPAI infection in ferret nasal turbinates

**Figure 2:**
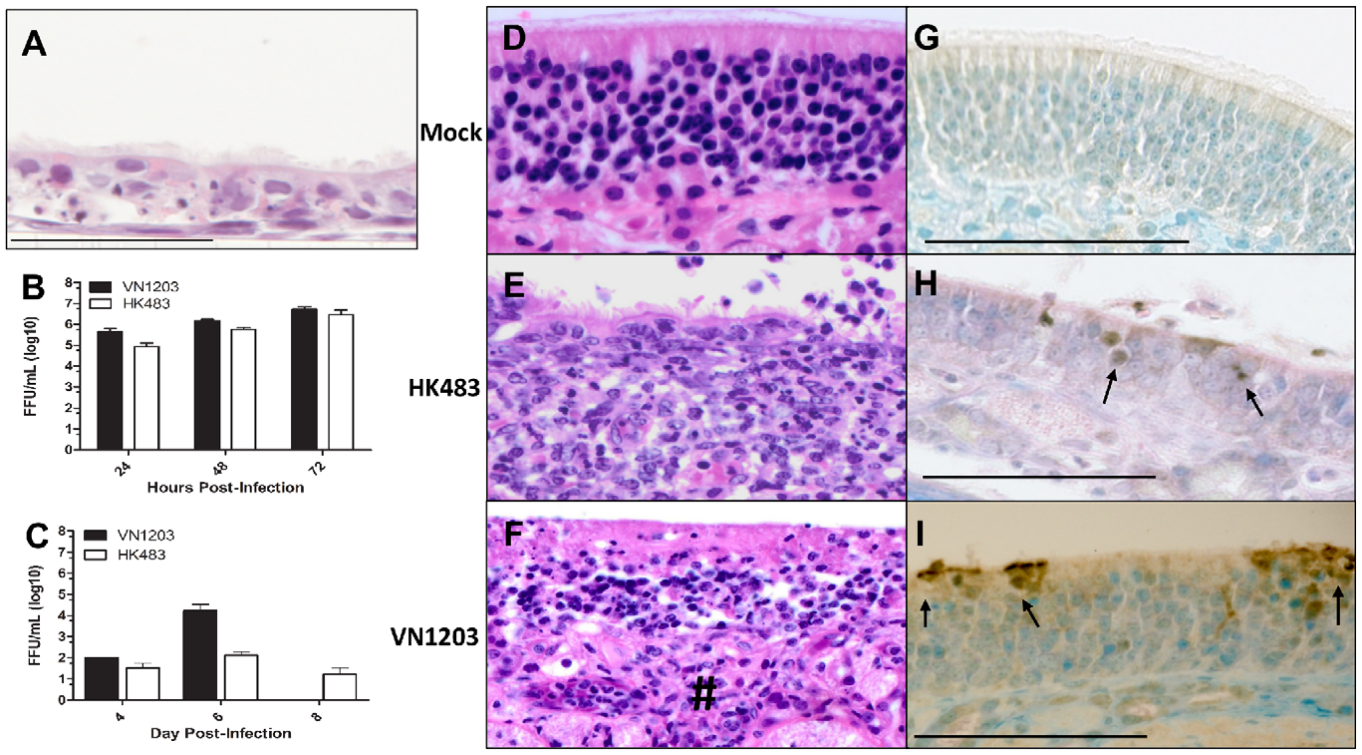
VN1203 induced significant pathology despite similar nasal turbinate titers in ferrets infected with either virus. **A.** H&E stained section a primary differentiated ferret nasal turbinate cell monolayer. **B.** Primary differentiated ferret nasal turbinate cells were infected with VN1203 or HK483 at an MOI of 0.001. Mean ± SEM of N = 4 samples per virus at each time point. **C.** Viral titers of homogenized nasal turbinates were graphed as mean ± SEM for VN1203 or HK483 infected ferrets. **D–I**. Representative samples of nasal turbinate tissue of ferrets instilled with allantoic fluid (**D,G**), HK483 (**E,H**), or VN1203 (**F,I**). **D–F** were stained with H&E, **G–I** were stained with anti-avian influenza NP and counterstained with Luxol fast blue. **F.** # indicates inflammatory cells infiltrating the underlying stroma. **H,I.** Arrows point to H5N1 infected cells. Scale bars: **A.** 50 µm; **D–F**, 300 µm; **G–I**, 100 µm.

Numerous reports suggest that virulence may be determined by replication kinetics in epithelial cells of the respiratory tract [20], [22]–[26]. To determine if increased replication in the nasal epithelium contributed to the increased lethality of VN1203 in ferrets, primary differentiated ferret nasal turbinate cells were isolated from naïve ferrets, cultured, and subsequently infected with each H5N1 strain. In preliminary studies, isolated nasal turbinate epithelial cells grown at an air-liquid interface developed a characteristic polarized, pseudostratified morphology and were able to maintain transepithelial electrical potential and an air-liquid barrier for greater than two weeks following culture (data not shown). The appearance of multiple ciliary axonemes by microscopy and visible production of mucus are indicative of differentiated cultures similar to normal human bronchial epithelial cells that are routinely used in this laboratory (**Figure 2A**). Ferret nasal turbinate cultures were infected at a multiplicity of infection of 0.001 of VN1203 or HK483 to simulate the low inoculating titer of H5N1 infected ferrets *in vivo*. Viral titers were measured from the apical wash at 24, 48, and 72 hours post infection and infection followed the characteristic replication kinetics observed for differentiated pulmonary epithelia as previously published by our group [26]. As shown in **Figure 2B**, there was no significant difference (ANOVA, F(1,6) = 1.88, p = .22) in the viral replication of these two HPAI strains, suggesting that increased replication in this cell type is not a key contributor to pathogenesis in ferrets.

To further assess viral replication in the upper respiratory tract, viral load was measured in the nasal turbinates of ferrets infected with VN1203 or HK483. Nasal turbinate tissue was isolated from ferrets undergoing planned necropsies at 4 and 6 dpi and homogenized for analysis by an immunostaining foci-forming assay. Similar viral titers were measured in the nasal turbinates of HK483 and VN1203 challenged ferrets at 4 and 6 dpi (Student's t-test, p = .42) (**Figure 2C**). Further examination of viral load in the nasal turbinates was performed by H5N1-specific qRT-PCR analysis of RNA isolated from homogenized nasal turbinate tissue. PCR standards of RNA genomic copy numbers were used to determine the gene copy numbers of infected ferret nasal tissues. Consistent with the findings from viral titer studies, the viral gene copy number was not different between VN1203 and HK483 at either 4 or 6 dpi, and indicate that viral load of VN1203 and HK483 are similar in the nasal tissues of ferrets (data not shown).

Tissue sections of formalin-fixed, paraffin-embedded nasal turbinates from VN1203 or HK483 infected ferrets were examined by microscopy for histological changes and infection with HK483 produced focal areas of ulcerations of nasal epithelium and underlying tissue (**Figure 2E**). Ulcerations were observed in approximately 10% of each nasal cavity examined; whereas ulcerations and erosion was observed in approximately 95% of VN1203 infected nasal cavities. The epithelial barrier remained largely intact though inflammatory cells infiltrated the underlying stroma. The inflammatory response was more pronounced in HK483 infection compared to VN1203 infection where inflammatory cell infiltration was not frequently observed. The nasal cavity of animals challenged with HK483 and examined 4, 6, and 8 dpi had fewer areas of ulceration in sensory epithelium, the sinuses, and epithelium lining the perimeter of the nasal cavity. In VN1203 infection, turbinate tissues showed larger and more numerous focal regions of epithelial changes (**Figure 2F**), although areas of normal appearance were present. Areas of histological changes were observed in more distal nares. Epithelial disruption and a discontinuous apical layer were characteristic of more severe regions with a loss of cellularity in the basolateral layer (**Figure 2F**). The presence of predominantly neutrophils in the submucosa was noted, as was neutrophil exudates in the nasal lumen. In HK483 infection, alterations of the nasal turbinates were milder at 4 dpi and increased by 6 and 8 dpi whereas histopathology of VN1203 were more numerous at 3 and 4 dpi. The distribution and severity of epithelial damage was milder in HK483 compared to VN1203 at all directly comparable time points. There was a pronounced acute inflammatory response associated with HK483 infection but was not a prominent feature in VN1203 infection.

Immunohistochemical analysis of influenza A NP (**Figure 2H,I**) indicated scattered focal areas of virus antigen detection in the olfactory neuroepithelial cells and in other cells in the intact epithelium of H5N1 infected ferrets as determined by the brown staining against the Luxol fast blue counterstain. The distribution and number of sites of virus expression in the nasal turbinates were variable between animals. Neuroepithelial staining exhibited a clustered appearance (**Figure 2I**) and this clustered staining was more apparent with VN1203 than HK483 infected nasal turbinates (**Figure 2H**).

### Pathological observations and viral loads in the lung of HPAI infected ferrets

**Figure 3:**
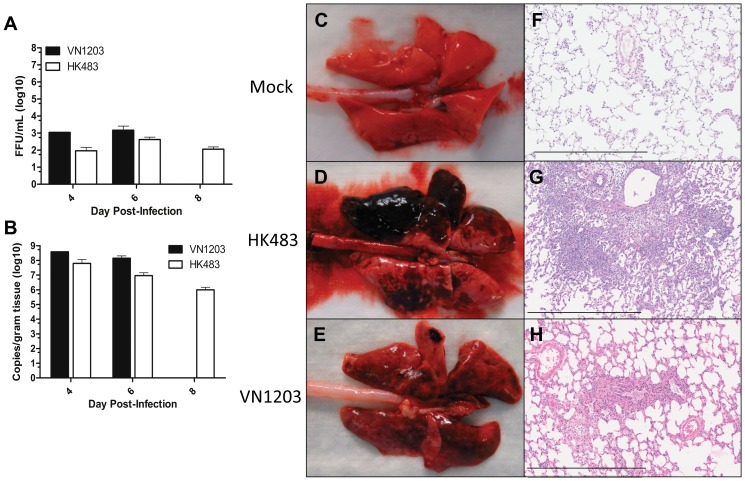
Viral titers and histopathology were similar in the lung of H5N1 infected ferrets. **A.** Viral titers and **B.** viral RNA was measured from four sections of the lung per ferret. **A,B**) Data represents the mean ± SEM from four lung regions of N = 1, VN1203 4 dpi, N = 3, VN1203 6 dpi, N = 6, HK483 4, 6, and 8 dpi. Dorsal view of ferret lung following intranasal instillation with allantoic fluid (**C**), HK483 (**D**), VN1203 (**E**). Lung sections stained with H&E of ferrets instilled with allantoic fluid (**F**), HK483 (**G**), VN1203 (**H**). **F–H**) scale bars = 600 µm.

To assess the extent of respiratory infection concurrent with the lethality and neurological signs of infection, viral titers from homogenized lung tissue were determined by an immunostaining foci-forming assay in ferrets at 4 and 6 days following infection. High viral titers were measured in the lungs of infected ferrets at 4 and 6 dpi, and viral titers in the lung were similar between ferrets infected with HK483 or VN1203 (**Figure 3A**). Additionally, there was no significant difference in the amount of viral RNA measured in the lung at 4 and 6 dpi for ferrets infected with either virus (**Figure 3B**). Consistent with the findings from nasal tissues, VN1203 and HK483 titers were not different in the lungs of infected ferrets.

Gross examination of lungs from HK483 or VN1203 infection was examined to assess infection-mediated lung disease in both HK483 and VN1203 infected ferrets. Large areas of dark reddish-purple consolidation were observed (**Figure 3D,E**) as compared to mock infected ferrets (**Figure 3C**). To investigate the histopathological changes in the lung of ferrets infected with VN1203 or HK483, randomized sections from four animals per group were stained with hematoxylin and eosin (H&E). Ferrets instilled with HK483 had few focal scattered areas of minimal to mild alveolitis and bronchiolitis on 4 dpi. Low numbers of epithelial cells detached into the lumen of alveolar spaces and there was infiltration of low numbers of neutrophils. There were peribronchiolar and scattered areas of alveolar parenchyma loss and infiltration of moderate numbers of neutrophil and mononuclear inflammatory cells 6 dpi. Locally extensive areas of bronchiolitis and peribronchiolar pneumonia, with localized loss of alveolar spaces in some animals were also observed (**Figure 3G**). The lung of ferrets challenged with VN1203 had multiple focal areas of pronounced cytopathology in the alveolar lining epithelium on 3 dpi. The severity of epithelial loss and damage to the interalveolar wall resulted in fibrin and edema exudate and hemorrhage in these areas. There was infiltration of low numbers of neutrophil inflammatory cells, primarily in the interalveolar wall. Examination of lungs at 6 dpi showed multifocal areas with moderate neutrophil infiltration in the alveolar walls and alveolar spaces and minimal to mild edema exudate (**Figure 3H**). While the type of damage induced by each strain was distinct, the distribution of lung damage in the airways and alveoli were equivalent following either VN1203 or HK483 infection in ferrets.

### HPAI viral load in the brain

**Figure 4:**
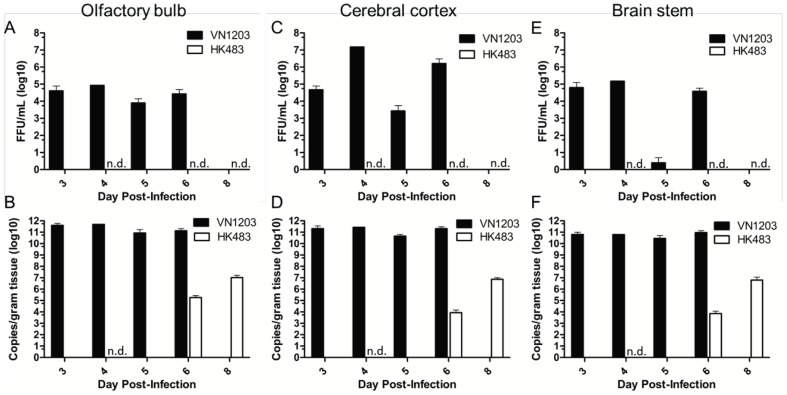
High viral titers were measured in the brain of VN1203 infected ferrets. **A,C,E**) Viral titers were measured in three sections of the ferret brain. Titers were graphed as mean ± SEM for VN1203 or HK483 infected ferret olfactory bulb (**A**), cerebral cortex (**C**), brain stem (**E**). Viral RNA was extracted from homogenized tissue and graphed as the mean ± SEM of viral RNA copies per gram of tissue from VN1203 or HK483 infected ferrets olfactory bulb (**B**), cerebral cortex (**D**), brain stem (**F**). White bars: HK483 infected ferrets, black bars: VN1203 infected ferrets. All mock infected ferrets were negative for virus. n.d. indicates virus or viral RNA was not detected. VN1203: N = 4, 3 dpi; N = 1, 4 dpi; N = 2, 5 dpi; N = 3, 6 dpi. HK483: N = 6; 4, 6 and 8 dpi.

Little is known regarding CNS involvement in the progression of HPAI disease. To determine the extent to which VN1203 and HK483 replicated in the olfactory bulb, tissue homogenates of the olfactory bulb were evaluated for viral titers. High viral titers were measured in the olfactory bulb of ferrets 3 to 6 days post challenge with VN1203. In contrast, no virus was recovered at 4, 6, or 8 dpi from the olfactory bulb of ferrets infected with HK483 (**Figure 4A**). To further assess more distal brain regions, viral titers from the cerebral cortex were also measured. Titers up to 10^7^ foci forming units (FFU)/mL were detected in the cerebral cortex of ferrets that died of VN1203 infection while no virus was recovered from HK483 infected ferrets (**Figure 4C**) examined at the peak of infection. VN1203 infected ferrets had approximately 10^5^ FFU/mL in the brain stem and viable virus was below the limit of detection (1 FFU/mL) in this tissue from ferrets infected with HK483 (**Figure 4E**). Typically, VN1203 was found at high levels in all animals at the time of death or euthanasia. In addition to high titers of viable VN1203 isolated from the brain sections, high copy numbers of viral RNA were also measured in the olfactory bulb, cerebral cortex, and brain stem of infected ferrets 3 to 6 days post challenge. Viral RNA copies were below the limit of detection in the brain of HK483 infected ferrets at 4 dpi and significantly lower than those measured from VN1203 infected ferrets at later time points (**Figure 4B,D,F**, ANOVA, p<.01). Collectively, these data indicate that VN1203 replicated to a higher titer in the brains of ferrets compared to HK483 at similar time points following infection and HK483 entry into the brain was delayed and less severe compared with VN1203.

### Localization of HPAI infection in the brain

**Figure 5:**
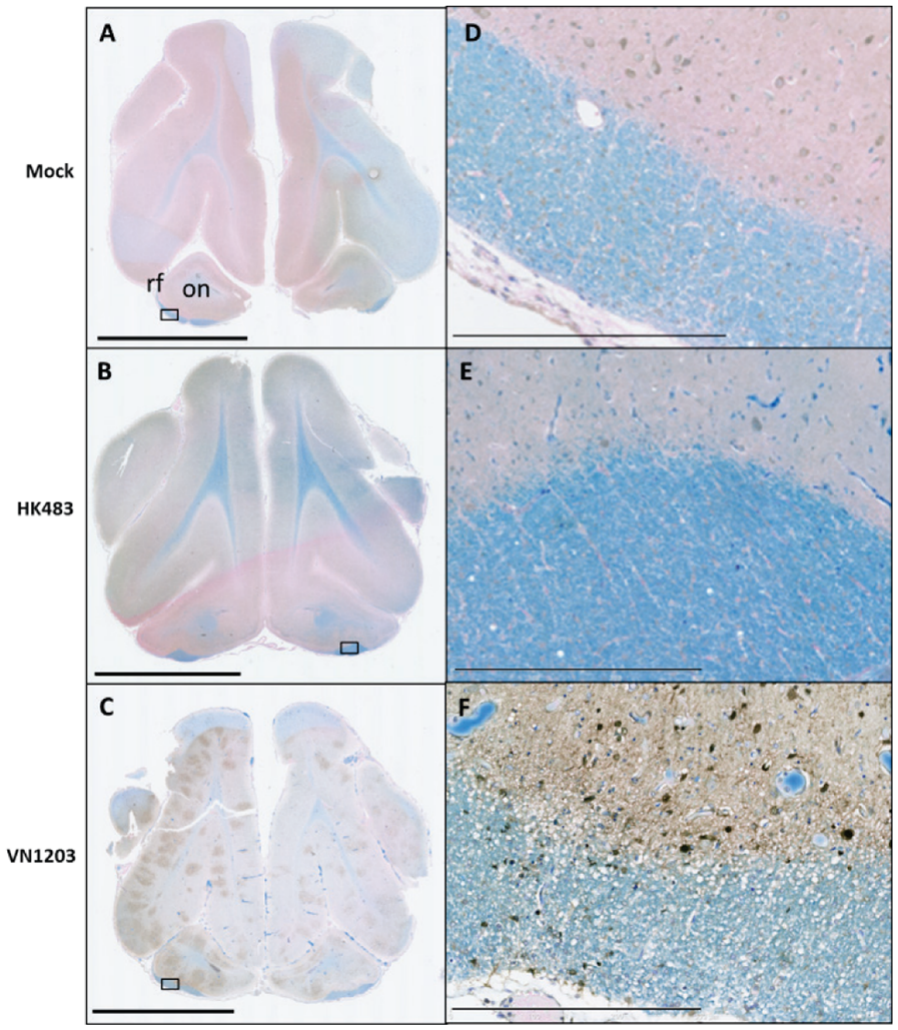
VN1203 infection in the ferret brain was multifocal and evident in the olfactory tract. Representative sections from the frontal lobe of ferrets intranasally instilled with allantoic fluid (**A,D**), on: olfactory nucleus, rf: rhinal fissure, HK483 (**B,E**) or VN1203 (**C,F**) were stained for viral antigen with IMGENEX 5187-A for avian influenza A NP and visualized with DAB. Brown stain indicates the presence of virus. Counterstained with Luxol fast blue. Scale bars: **A–C**) 5 mm, **D–F**) 300 µm.

Routes of infection in the CNS have been suggested [10]–[12], [21], [27]; however, the dissemination of virus in the brain has not been clearly elucidated. To localize VN1203 and HK483 antigen in the brain of ferrets, multiple 5 µm sections of the frontal cortex, cerebrum, and cerebellum were obtained from formalin-fixed, paraffin-embedded brain tissues and examined by immunohistochemistry for avian influenza NP. Normal morphology was observed in the frontal cortex of mock-infected ferrets (**Figure 5A**) and HK483 NP antigen could not be detected from any brain section of ferrets challenged with HK483 and examined 4 dpi (**Figure 5B and 5E**). A single focus of staining was observed in the olfactory nucleus of one ferret each at 6 and 8 dpi (data not shown). Strikingly, numerous distinct foci of VN1203 NP expression were detected 3 dpi in the olfactory bulb and frontal cortex (**Figure 5C**) and similar expression was also observed 4 to 6 dpi. Foci were widespread and contained numerous nuclear-stained cells (**Figure 5F**).

**Figure 6:**
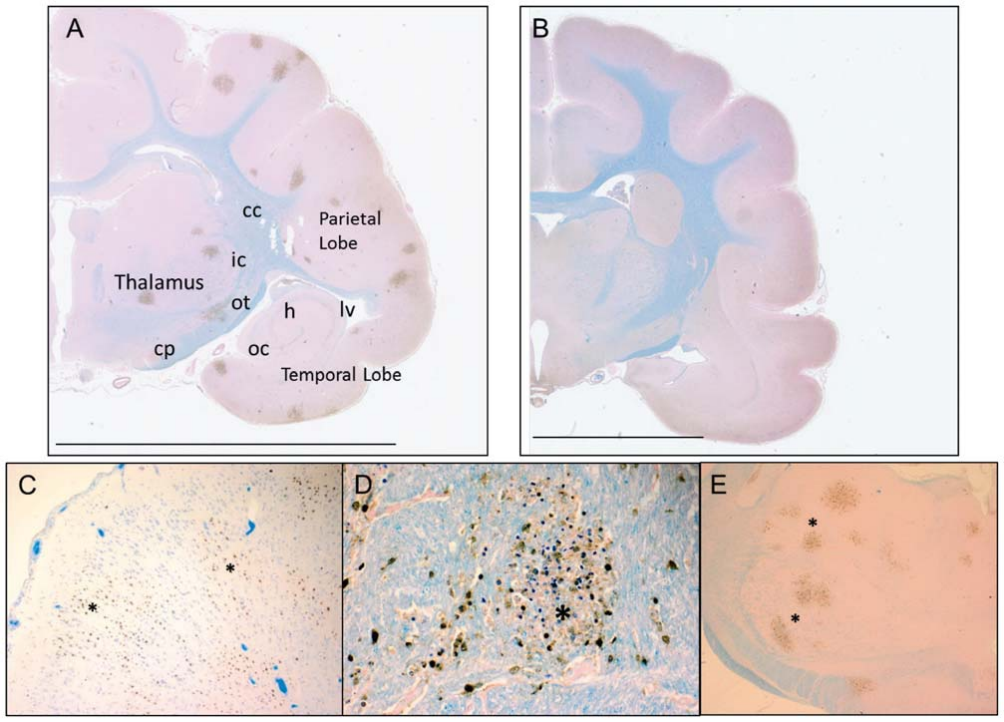
VN1203 infection resulted in multifocal infection in the cerebral cortex of ferrets. Representative sections of the cerebrum of ferrets intranasally instilled with VN1203 (**A, C–E**) and HK483 (**B**). Viral antigen was detected with IMGENEX-5187A for avian influenza A NP and visualized with DAB. Brown stain indicates the presence of virus. Counterstained with Luxol fast blue. Scale bars represent 10 mm. cc: corpus collosum, ic: internal capsule, ot: optical tract, h: hippocampus, lv: lateral ventricle, oc: olfactory cortex, cp: cerebral peduncle.

Similar to observations in the frontal cortex, multiple foci of VN1203 were identified 3 dpi in the occipital cortex of the temporal lobe, the ectorhinal cortex, striatum, somatosensory area of the parietal lobe, the internal capsule, and the adjacent thalamus (**Figure 6A**). In contrast, no viral antigen was detected in these regions of ferrets infected with HK483 and examined at 4, 6, or 8 dpi (**Figure 6B**). Foci of VN1203 were numerous, widespread, and concentric in appearance. Furthermore, virus was also identified in the dorsal cerebral cortex where no histologic alterations or inflammatory cell infiltration was observed (**Figure 6C**). VN1203 was observed in the internal capsule (**Figure 6D**) and the adjacent thalamus (**Figure 6E**), representing new areas of infection that have not been described previously. Foci in these regions were similar to those in other brain regions.

**Figure 7:**
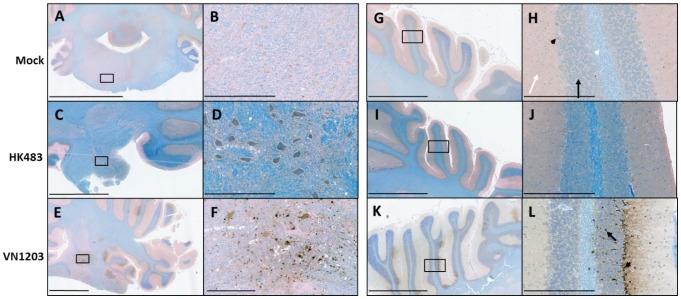
VN1203 infected and replicated in Purkinje cells and deep cerebellar nuclei of the cerebellum. Representative sections of the cerebellum of ferrets instilled with allantoic fluid (**A,B,G,H**), HK483 (**C,D,I,J**), VN1203 (**E,F,K,L**). Viral antigen was detected with IMGENEX 5187-A for avian influenza A NP and visualized with DAB. Brown stain indicates the presence of virus. Counterstained with Luxol fast blue. (**B–F**) higher magnification of boxes in A–E respectively. H) White arrow indicates molecular layer, black arrow indicates granule layer, black arrowhead indicates a Purkinje cell, white arrowhead indicates Purkinje cell axons. **L**) Arrow indicates infected granule cell and arrowhead indicates infected Purkinje cell. Scale bars represent: **A,C,E,G,I,K**) 2 mm; **B,D,F,H,J,L**) 300 µm.

Hindbrain tissue sections were obtained from infected ferrets and assessed for HK483 and VN1203 at multiple time points. Hindbrain regions included the cerebellum, pons, medulla, and the most rostral aspects of the spinal cord (**Figure 7A**). Hind rains from HK483-inected ferrets appeared normal with an absence of any immunohistochemical staining for influenza NP at any time during the infection period and concurrent with active infection in the respiratory tract (**Figure 7C,D,I,J**). In contrast, viral antigen was observed in multiple regions of the brain stem, cerebellar peduncles, and cerebrobulbar tracts in white and gray matter areas. More specifically, multiple foci of infection were identified in the Purkinje cells of the cerebellar foci (**Figure 7K,L**) with occasional staining extending into the granular layer, which is penetrated by Purkinje cell axons. Interestingly, the adjacent molecular layer that consists of dendrites extending from the Purkinje cells exhibited a complete lack of VN1203 staining, suggesting directional migration of VN1203 at least in the cerebellar folia. The widespread dissemination of VN1203 suggests that the virus did not spread from a single dissemination site.

### Brain lesions during HPAI infection

**Figure 8:**
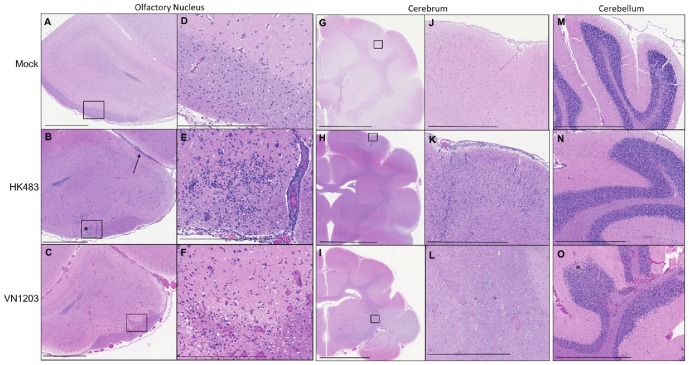
VN1203 resulted in severe and widespread brain lesions compared with HK483. Representative sections from the olfactory nucleus (**A–F**), cerebrum (**G–L**), and cerebellum (**M–O**) were taken from ferrets instilled with allantoic fluid (**A,D,G,J,M**), HK483 (**B,E,H,K,N**), VN1203 (**C,F,I,L,O**) and stained with H&E for evaluation of histological alterations. **B**) Arrow points to meningeal inflammatory cell infiltration, asterisk denotes infiltration of neutrophils. **D–F**) High magnification of boxes in A–C respectively. **O**) Asterisk denotes lesion. Scale bars: **A–C, J–L, M–O**) 1 mm, **D–F**) 300 µm, **G–I**) 5 mm.

Tissue sections from the forebrain, midbrain and hindbrain were randomly selected, stained by hematoxylin and eosin and examined under light microscopy for evidence of histological changes related to viral neuroinvasion. In contrast to ferrets infected with VN1203 in which brain lesions were present on 3 dpi, there were no histological alterations in the brains of ferrets infected with HK483 examined on 4 dpi. Two of six HK483 infected animals examined on 6 dpi had acute neutrophil inflammatory cell infiltration in the olfactory system located in the olfactory nuclei in the frontal cortex (**Figure 8B,E**), the olfactory cortex in the temporal lobe, limbic system, and the somatosensory area in the parietal lobe. One of the two ferrets with observed inflammatory cell infiltration had viral antigen present in the olfactory nuclei while it was not detected elsewhere in the brain (data not shown). Two of six HK483 infected animals examined 8 dpi had more advanced mild to moderate acute inflammation limited to the olfactory system. Likewise, immunostaining was observed in only the olfactory nucleus in one HK483 infected ferret 8 dpi. Meningoencephalitis was pronounced in the olfactory system in a HK483 infected ferret that had lost more than 25% of its initial body weight, and had no neurological signs when examined 11 dpi.

Histological examination of ferrets infected with VN1203 on days 3–6 post challenge showed widespread lesions in gray and white matter areas in the frontal cortex (**Figure 8C,F**), thalamus, parietal and temporal lobes, midbrain/medulla (**Figure 8I,L**), and hind brain cerebellum folia (**Figure 8O**) in eight of ten animals, in sharp contrast to the brains of ferrets infected with HK483 where lesions were not observed (**Figure 8B,E,H,K,N**). **Figure 8A, D, G, J, and M** demonstrate healthy mock infected brain sections of ferrets. Low numbers of neutrophil infiltration in the olfactory bulb, and areas of cell death and/or loss of cellularity were evident in all brain regions of VN1203 infected ferrets. Microglial nodules were observed in regions near influenza foci (**Figure 8C,F**). Furthermore, inflammation of the meninges was evident in the frontal cortex consisting of increased cellular infiltration and increased size of the arachnoid space (**Figure 8C**). Lesions observed in the cerebrum and cerebellum of VN1203 infected ferrets corresponded to regions of viral antigen detection by immunohistochemistry while HK483 infected ferrets did not have lesions or viral antigen detected in the cerebrum or cerebellum.

## Discussion

Although neuropathogenesis and neurological sequelae have been recognized for HPAI infection, little is known about the contribution of neuroinvasion to lethality against the well-studied role of respiratory disease during influenza infection. Here we show that low infectious titers of distinct H5N1 HPAI viruses produce divergent disease outcomes amid distinct neurovirulence. We also show that neurological signs are important indicators of replicating virus in the brain and likely foretell morbidity and lethality in the ferret model. Infection with the clade I prototypic strain VN1203 at low inoculating titers led to substantial lethality and neurological signs, whereas the clade 0 prototypic strain, HK483, resulted in less lethality and no neurological sequelae. While cells of the lower respiratory tract are often the primary targets of influenza A virus, nasal turbinate cells including neuroepithelial bundles of the olfactory tract are also permissive to infection. In primary differentiated ferret nasal turbinate cells in culture, VN1203 and HK483 replicated to similar titers consistent with data obtained from the nasal turbinates of VN1203 or HK483 infected ferrets. Likewise, while the damage caused by each virus was distinct, the viral replication was similar in the lungs of ferrets infected with either VN1203 or HK483 suggesting that productive infection in the respiratory tract was an insufficient predictor of lethality in this model.

Remarkably, VN1203 infection was multifocal and widespread throughout the brain while HK483 was much less neurotropic with lesions limited to the olfactory system. The absence of neurological clinical signs in ferrets infected with HK483 correlated with histopathological immunostaining that demonstrated virus was limited to the olfactory system. The restriction of virus primarily to the olfactory nucleus and olfactory cortex also correlated with the low viral RNA copy number in the brain. Viral RNA was detected in the olfactory bulb of approximately 75% of animals 6 and 8 dpi. All HK483 infected animals were negative on 4 dpi showing a delayed time course of transmission of virus to the olfactory bulb and brain in ferrets infected with HK483 compared to VN1203. These findings suggest differences in pathogenesis and neurotropism that correlated with the extent of dissemination within the CNS and are possibly indicative of differences in viral replication in the CNS. The prominent histological damage in the central olfactory system in the brain at 6 and 8 dpi in ferrets infected with HK483 were associated with marked inflammatory cell infiltration and showed HK483 induced alterations associated with only low levels of viral RNA detected in the brain by qRT-PCR. Multiple brain regions (forebrain, midbrain, and hindbrain) and several tissues within each region were found to have active VN1203 replication.

HK483 and A/Hong Kong/486/1997 have been used in murine models of infection to elucidate routes of neuroinvasion, and from these studies, the trigeminal, vagus, and sympathetic nerves [11], [12], [27] were identified in addition to the olfactory nerves. In the present study, virus expression was detected in the trigeminal and facial nuclei in the brainstem of ferrets infected with VN1203. Fibers from these nuclei are distributed in the reticular activating system and enter the cerebellum through the cerebellar peduncles. They also project to the thalamus and connect to the motor cortex via the corticobulbar tract. Ferrets had clinical signs of ocular discharge indicative of conjunctivitis, a possible site of entry into the terminal tributaries of the trigeminal nerve. The hypoglossal nerve innervates the tongue and pharynx and the nucleus in the brainstem receives efferents from the sensory trigeminal nuclei. The data presented here indicate that the olfactory nerves may be an important conduit of neuroinvasion; however, other routes are plausible based on our findings of VN1203 replication and pathogenesis in multiple brain regions.

Other viruses such as JC virus, poliovirus, Epstein-Barr virus, mouse adenovirus 1, human T-lymphotropic virus type 1, and West Nile virus are known to infect the CNS through the vascular endothelium [28]. HPAI has not been shown to enter the CNS through this route and virus was not detected in the CNS endothelium of the ferrets infected with VN1203 or HK483 in this study. A more likely method of CNS entry is through the peripheral nerves. It has been shown that poliovirus, adenoviruses, and rabies bind to neurons at the neuromuscular junction where specific receptors are expressed [28]. While influenza receptors on neurons are not known, Jang *et al* [29] used microfluidic chambers to show VN1203 could be transported from the processes of freshly dissociated dorsal root ganglia cells of mice to the cell body in a separate compartment. This finding demonstrated that VN1203 traveled via the axons suggesting a method for transport of H5N1 to the CNS. Here, the distribution of virus antigen throughout various regions of the brain indicates that VN1203 may have entered the brain via multiple cranial nerves though the mechanism remains to be elucidated.

The infection of the olfactory region may be particularly important in the widespread infection observed in multiple brain regions prior to death. Cell death and ulceration of sensory epithelial cells with clinical signs of nasal discharge and viral expression strongly suggest that the nasal infection was the route of entry into the olfactory bulb and for subsequent dissemination in the central olfactory nervous system of both stains of virus. Influenza has been shown to infect olfactory nerves [18], [19], [27] and immunostaining demonstrated virus expression in areas in the cerebrum in which mitral cells project from the olfactory bulb to synapse in the anterior olfactory nucleus, the olfactory cortex in the temporal lobe, the amygdala, the piriform cortex, and the enterhinal cortex. Multiple projections from the olfactory bulb may explain some of the widespread viral antigen observed in the cerebral cortex. The olfactory system is the only cranial sensory system that has direct projections into the cerebral cortex without relay in a thalamic nucleus. Also, the close neuroanatomical connections of the olfactory system to the limbic system help explain VN1203 distribution in the hippocampus and the striatum.

The nature of the neurological signs following VN1203 infection may have important implications for clinical evaluation of active H5N1 infection. Animals that succumbed to VN1203 infection at 3 dpi exhibited convulsions leading up to death. Consistent with these findings, VN1203 was isolated from the cerebral cortex from all animals that exhibited these signs. Further evidence of the link between viral localization and specific neurological signs can be observed in the later phase of lethality to VN1203. Ferrets that succumbed to infection at 5 or 6 dpi exhibited a lack of voluntary movement control and coordination. Upon analysis of viral localization in the brain, virus was isolated from the deep cerebellar nuclei, an area known to be important in motor control and gait. One important finding from the current studies is the elucidation of Purkinje cells of the cerebellar foci as a strongly permissive cell for H5N1 infection. Likewise, the granular layer, a region that receives axons from the Purkinje cells, but not the molecular layer, which consists of dendrites from Purkinje cells, was found to have substantial viral antigen staining and may indicate directional migration of H5N1 in neuronal tissues. Although further study is warranted, the notion that specific neurological signs may be used to ascertain the presence of virus in various brain regions may become useful should therapeutics targeting neurological infection become available.

The neurovirulence and mechanisms of brain pathogenesis of HPAI H5N1 are not known and require further study. Clearly, viral determinants exist in different H5N1 viruses that lead to neurotropism in ferrets. Further studies to elucidate the mechanism by which H5N1 enters into and is disseminated throughout the brain will be important for identifying H5N1 viruses through global surveillance that may have enhanced pathogenesis in humans. Additionally, the route of infection and dissemination within the brain should be more clearly defined to provide clinicians with more markers of brain involvement in H5N1 infection and to identify novel areas as targets for therapeutics to prevent virus dissemination to the CNS. It is also unclear whether entry routes along different cranial nerves require specific receptors for viral entry, or if it is opportunistic in nature. Furthermore, therapeutic approaches that target influenza in the brain will likely need investigation in light of the rapid onset of neurological signs in human cases. While not widely appreciated outside of the influenza research community, the importance of neurological disease in HPAI should be emphasized in future public health initiatives.
